# DOPE++: 6D pose estimation algorithm for weakly textured objects based on deep neural networks

**DOI:** 10.1371/journal.pone.0269175

**Published:** 2022-06-08

**Authors:** Mei Jin, Jiaqing Li, Liguo Zhang

**Affiliations:** 1 School of Electrical Engineering, Yanshan University, Qinhuangdao, China; 2 Hebei Key Laboratory of Testing and Metrology Technology and Instruments, Yanshan University, Qinhuangdao, China; National University of Sciences and Technology (NUST), PAKISTAN

## Abstract

This paper focuses on 6D pose estimation for weakly textured targets from RGB-D images. A 6D pose estimation algorithm (DOPE++) based on a deep neural network for weakly textured objects is proposed to solve the poor real-time pose estimation and low recognition efficiency in the robot grasping process of parts with weak texture. More specifically, we first introduce the depthwise separable convolution operation to lighten the original deep object pose estimation (DOPE) network structure to improve the network operation speed. Second, an attention mechanism is introduced to improve network accuracy. In response to the low recognition efficiency of the original DOPE network for parts with occlusion relationships and the false recognition problem in recognizing parts with scales that are too large or too small, a random mask local processing method and a multiscale fusion pose estimation module are proposed. The results show that our proposed DOPE++ network improves the real-time performance of 6D pose estimation and enhances the recognition of parts at different scales without loss of accuracy. To address the problem of a single background representation of the part pose estimation dataset, a virtual dataset is constructed for data expansion to form a hybrid dataset.

## 1. Introduction

Image-based 6D target pose estimation [1] is playing an increasingly important role in applications such as virtual reality [2–4] and robotics operations [5–7]. 6D pose estimation refers to finding the image object position and calculating the rotation translation relationship between the object coordinate system and the camera coordinate system. 6D refers to the 3D position and 3D pose of an object, and its physical quantities are the three translational and three rotational parameters of the object. The significance of 6D pose estimation is to accurately obtain the pose of an object to support the fine manipulation of the object [8] and it is mainly used in industrial scenarios such as cargo sorting and robot grasping. Robots using a 6D pose estimation algorithm can further estimate their pose based on detection of target parts to more accurately grasp the parts [9]. In practical scenarios of industrial manufacturing, the core problem of robot gripping work is the accurate recognition of the 6D pose of an object. The traditional algorithm approach requires manual definition of 3D feature description, matching the point cloud of the object to be captured from the field scan with the known point cloud of the object model to be captured for 3D template matching, and convex optimization to reduce the error after alignment. Thus, a current research trend is to restore 6D pose information of objects based on texture features of a priori object models by inputting monocular RGB images [10, 11]. With the rise of RGB-D depth sensors, methods to combine the depth information acquired by the sensors with RGB images for robotic arm grasping tasks have also started to emerge [12–14]. Compared with a single RGB image for pose estimation, the depth sensor-based RGB-D image method can contain more geometric information about the target and perform more consistently and reliably in the pose estimation process [9]. Therefore, we focus on 6D pose estimation using a single RGB-D image.

Traditional pose estimation methods are mainly divided into keypoint-based methods [15–17] and template-based methods [18, 19]. The traditional method mainly constructs local feature descriptors (SIFT, HOG, ORB, etc.) of the target object, extracts feature points in the image, and constructs feature descriptors for feature matching. Then, the 6D pose of the object is calculated using the correspondence between 2D and 3D. However, the estimation effect is not satisfactory for objects with weak textures and obscure features, such as some mechanical parts for industrial use.

In recent years, with the rapid development of deep learning, convolutional neural network (CNN)-based pose estimation has become the mainstream method for pose estimation. Compared with traditional methods, CNN-based methods have strong resistance to complex environments, are more adaptable to common clustering methods in daily life [20], and perform well in the recognition of some weakly textured objects. CNN-based pose estimation methods are mainly classified into end-to-end and two-stage methods. End-to-end methods that directly regress the object pose, such as PoseCNN [14] proposed by Xiang et al. and SilhoNet [21] proposed by Billing et al., have difficulty in computing the rotation and translation of the target. Wang et al. proposed the DenseFusion algorithm [22], which uses RGB-D images as the input to the network while employing an end-to-end iterative refinement of the network to greatly improve the pose estimation accuracy. However, the end-to-end approach still faces the challenge of difficulty in estimating the translations of the target. The two-stage approach regresses the key points of the target using a CNN and then calculates the pose of the object by the Perspective-n-Point (PnP) algorithm [23]. YOLO-6D proposed by Tekin et al. [24] and BB8 proposed by Oberweger M and Hu y et al. [25, 26] both predict the projection of the object’s 3D minimum bounding box on the 2D picture and then calculate the 6D pose of the target by the PnP [23] algorithm, eliminating the practice of fine-tuning the correction of the result on previous methods. Kehl et al. proposed the SSD-6D algorithm [10], which first locates the 2D bounding box of the target and then matches it with the associated 6D pose. Nigam et al. proposed a novel network architecture [27] that combines local features of coordinate regression with global features to further improve the accuracy of 6D pose estimation. Jonathan Tremblay, Yu Xiang et al. proposed an algorithmic framework for six-degrees-of-freedom object pose estimation based on keypoint detection, namely, the DOPE (deep object pose estimation) [28] algorithmic framework. The algorithm takes an input RGB image and obtains image features by feature extraction and then obtains the confidence map and vector map of the image through a six-step pose estimation network, which innovatively converts the target detection task into a regression problem. The algorithmic framework innovatively inferred the 6D pose of a known object from a single RGB image without subsequent alignment.

**Fig 1:**
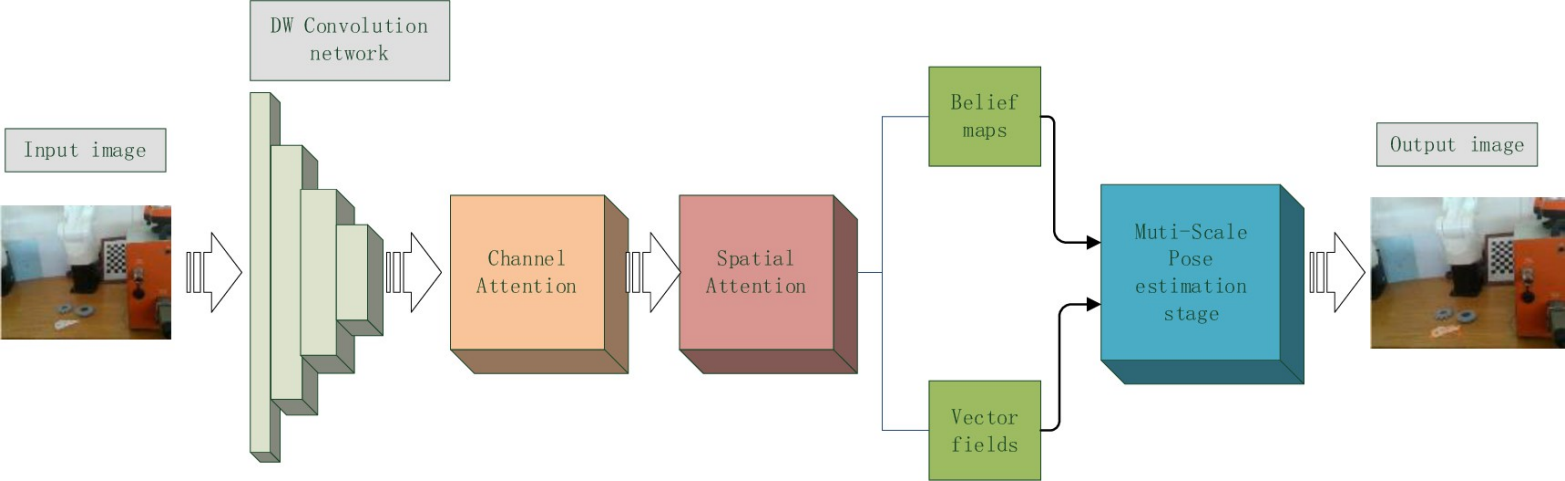
The overall framework of the DOPE++ network. The input image produces two branches, a confidence map and a vector map, after passing through DW convolution (a deeply separable convolutional network) and an Attention Mechanism. The two feature maps are fused and then import our proposed new Multi-Scale pose estimation module. Finally, the 6D poses of the target are obtained.

Although the 6D pose estimation algorithms in the last two years have been well addressed in terms of accuracy and efficiency, the DOPE algorithm proposed by Jonathan Tremblay, Yu Xiang et al. deserves another in-depth consideration in terms of network architecture and synthetic data. Therefore, we take the DOPE algorithm as a benchmark and focus on solving the problems of the original DOPE algorithm, such as the slow recognition of objects, difficulty in resisting large-scale changes in targets, and difficulty in accurately recognizing occluded targets using only RGB images, in our algorithm DOPE++. Alongside comparing our proposed network with the original DOPE network, we compared it with some state-of-the-art algorithms, such as the DRNet algorithm [20] and the DenseFusion algorithm [22]. Experiments demonstrate that our proposed model excels in both accuracy and efficiency of 6D pose estimation. Fig 1 shows the overall framework of the DOPE++ network.

The main innovations of this paper are as follows.

1. To improve the running speed of the network, the fusion model lightweighting method is improved for feature extraction, which improves the frame rate by 14 FPS compared to the original network, and the running time of the total pose estimation reaches 0.033 seconds per frame.
2. To reduce the loss of accuracy due to the reduction in the number of network parameters, an attention mechanism is introduced to improve the detection accuracy.
3. To cope with false detection and missed detection caused by the scale variation of the original network for the parts to be detected, a multiscale fusion of the pose estimation module is proposed to further improve the network accuracy.
4. To address the problem of part occlusion in engineering, this paper proposes a random mask local processing method to improve the dataset and optimize the accuracy of the network in dealing with object occlusion cases.
5. Most of the existing 6D pose estimation methods use datasets such as LineMOD [29] or YCB-Video [14], which lack weakly textured industrial parts. Thus, this paper proposes a method to produce datasets for weakly textured parts using virtual reality technology.
6. A vision-guided robot grasping platform is established to verify the feasibility of the proposed algorithm for manufacturing applications such as grasping and assembly.

## 2. Related work

In recent years, 6D pose estimation networks based on deep learning have excelled in terms of accuracy and efficiency [20, 29–31]. However, the lack of real data for training the network makes it difficult to expand the network to new application scenarios, such as in the field of smart manufacturing [32–34] and autonomous driving [35, 36]. For this purpose, we use virtual reality techniques [37–40] to produce datasets on weakly textured industrial parts. We independently design a series of comparative experiments to verify the advantages of using virtual reality technology to produce datasets, such as avoiding the problems of a single background, small changes in object position and pose, and easy overfitting that exist in real datasets of YCB videos [41, 42]. In addition, a random mask localization method is proposed for the part occlusion problem to optimize the accuracy of the network in dealing with object occlusion cases.

### 2.1. Virtual reality-based dataset production of weakly textured parts

**Fig 2:**
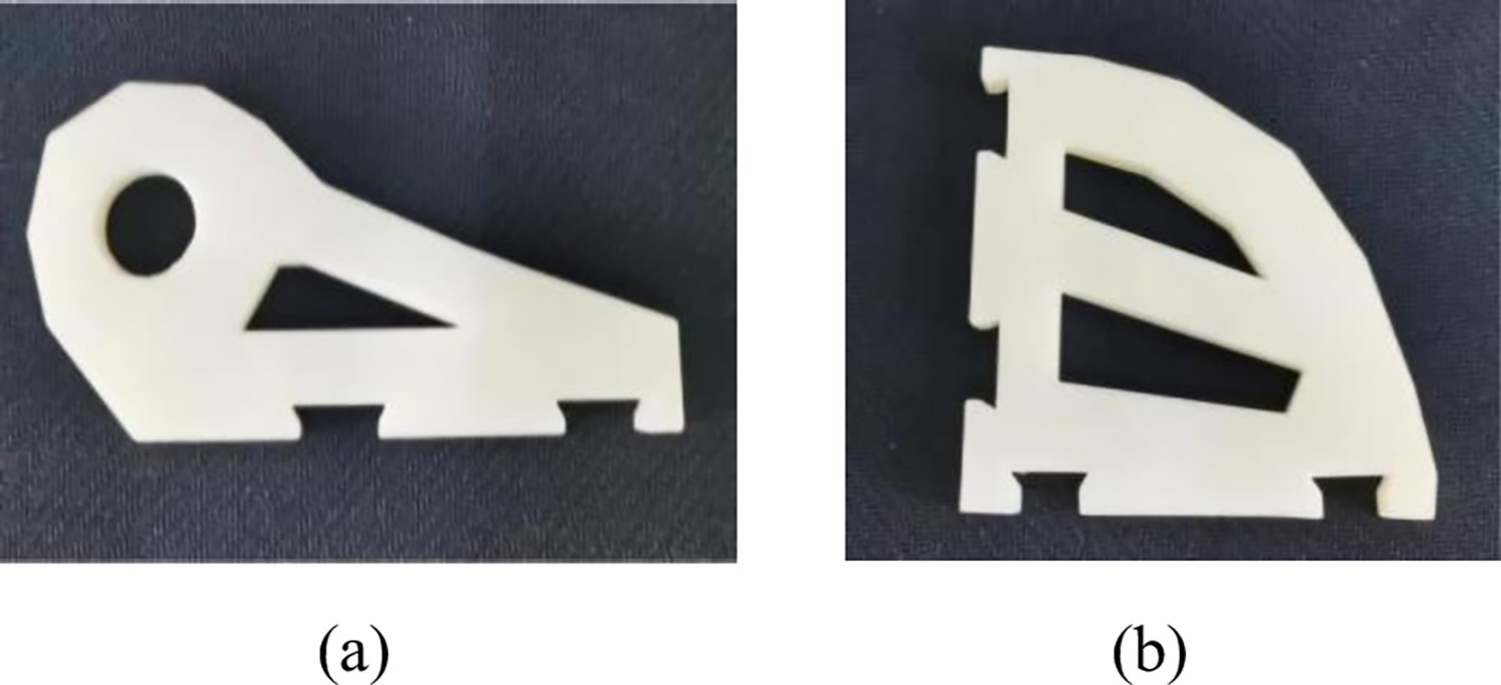
3D printed models of the weakly textured parts. (a)Servoholder. (b)PivConnector.

This study utilizes unreal engine 4 (UE4) as the device for generating virtual datasets. Specifically, the SolidWorks toolbox was first used to create a 3D model of the robot connections. The 3D printing results are shown in Fig 2.

**Fig 3:**
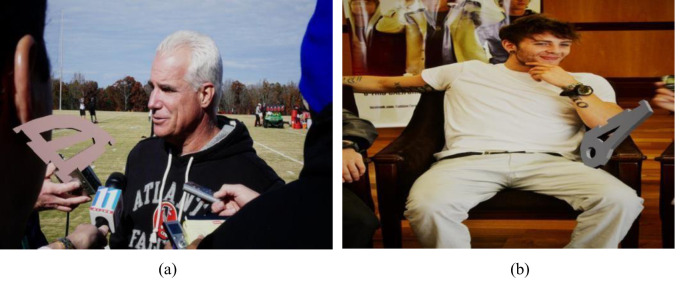
A dataset produced using virtual reality technology. (a) Example of a PivConnector dataset. (b) Example of a Servoholder dataset.

The Servoholder and PivConnector are arbitrarily selected as illustrations, where the size of the Servoholder is 7.7×7.7×0.7 (in cm) and the size of the PivConnector is 10.5×5.2×0.7 (in cm). The centre coordinates location and attitude rotation of the model need to be generated automatically after building the workpiece model. Compared with manual annotation, the UE4-based annotation method can use the complete geometric information in the constructed model to automatically calculate the object centre-of-mass coordinates to eliminate pixel errors and can define the relative positions of the camera and the workpiece to eliminate reprojection errors caused by misalignment. To solve the problem of object fixation, this paper sets the object to move randomly and rotate randomly. To solve the problem of a single background, we add a random background as interference for the object; the background image is from the VOC2007 dataset [43]. The original position is in the world coordinate system for the connector coordinates (0,0,0), the camera coordinates (-20,0,0), the camera distance from the connector is 20 cm with vertical shooting. The camera position is of a fixed resolution of 640×480. The sections of random movement in the x-axis is (-10,10), y-axis is (-15,15), and z-axis is (-15,15). The connector randomly rotates around the x-axis, y-axis, and z-axis. UE4 accurately marks the 6D pose of the object based on the object geometry information and the defined camera parameters. The effect of the generated dataset is shown in Fig 3.

The method of generating datasets using virtual reality allows setting the position and rotation of objects randomly. We also set the light angle and light intensity to random sizes and randomly replace the background texture to avoid overfitting to a specific data distribution.

### 2.2. Validating the advantages of using virtual reality to produce datasets

To verify the effectiveness of the dataset produced based on virtual reality, we illustrate a set of comparative experiments. The DOPE++ network is first trained on a hybrid dataset obtained by extending the virtual dataset produced using the model from the YCB-Video dataset. The comparison of metrics illustrates the superiority of training in the hybrid dataset over the real dataset. More specifically, objects numbered 003, 005, 006, 009, and 010 in the YCB-Video dataset [13] were randomly selected for the experiments. A commonly used evaluation criterion in object 6D pose detection is the 3D distance of the average model point (ADD, average distance) [13, 44]: the average distance deviation is calculated from the 3D model points under the estimated poses and the 3D model points under the true poses; the poses are considered to be correctly estimated if the deviation is less than 10% of the object diameter, i.e., the threshold value is 0.1 m. ADD is calculated as [27].

$$ADD=\frac{1}{n}\sum_{i=1}^{n}||(R_{est}P_{i}+T_{est})-(RP_{i}+T)|{|}_{2}$$ (1)

where *R*_*est*_ is the predicted rotation matrix, *T*_*est*_ is the predicted translation matrix, *R* is the rotation matrix of the true value, *T* is the translation matrix of the true value, and *P*_*i*_ is the 3D point in the object. When the ADD value is less than the threshold value, the pose estimation is considered correct and is called a true positive (TP), i.e., correctly detected pose; greater than this value, the pose estimation fails and is called a false negative (FN), i.e., incorrectly detected pose. The ADD values for each type of object at different thresholds are calculated, and the ADD pass rate [8] at different thresholds is given by

$$ADDpassrate=\frac{TP}{TP+FN}$$ (2)

**Fig 4:**
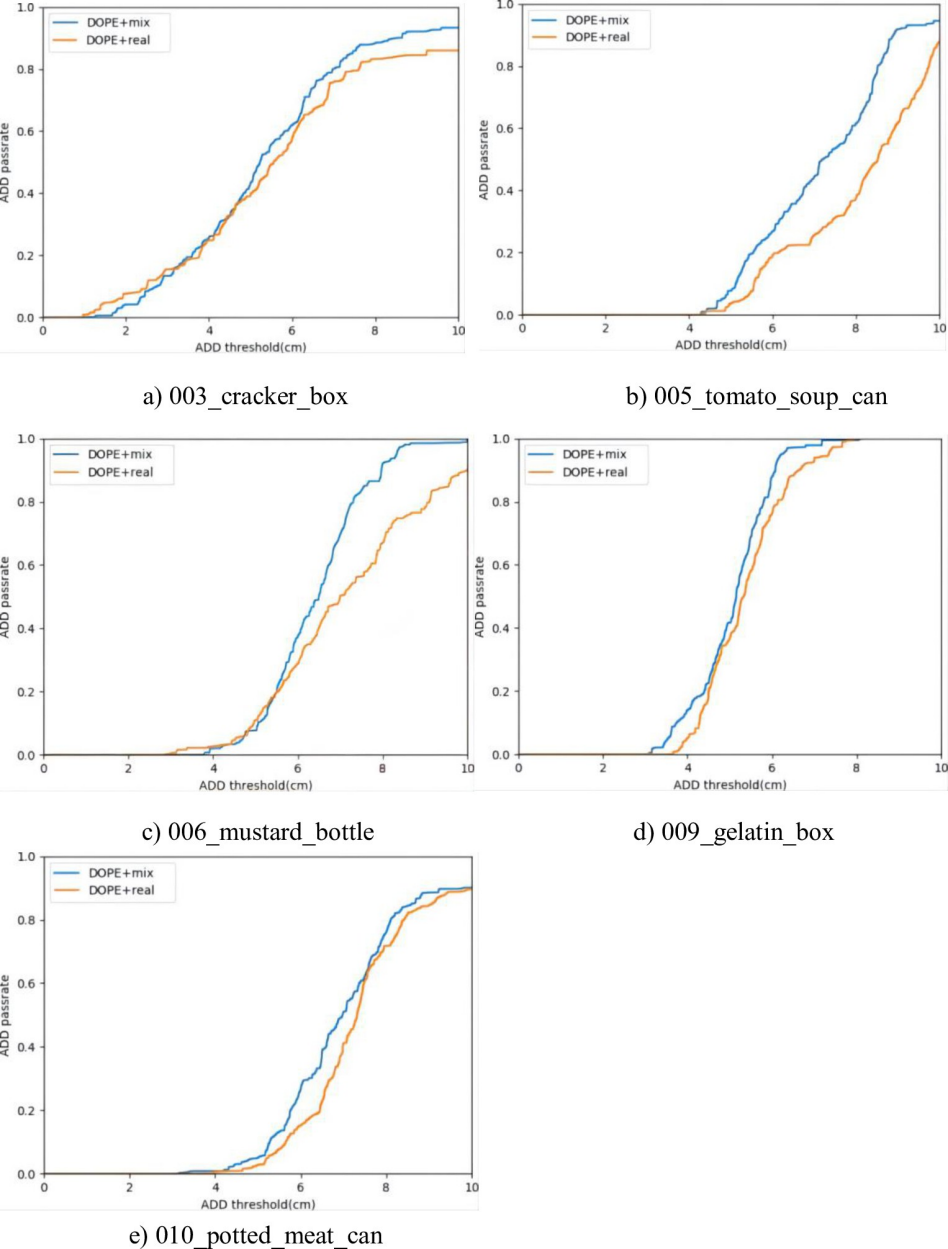
AUC plots for the five objects. Where the blue line indicates the training result of the network under the mixed data set and the yellow line is the result of the network trained under the realistic data set.

All results were plotted as a curve, and the ratio of the area under the curve to the total area was called the area under the curve (AUC) [27]. The AUC responds to the classifier’s ability to rank the samples. We use these two metrics to evaluate the hybrid dataset produced by using virtual reality technology. Table 1 shows the comparison of the training results of the DOPE++ network on the YCB-Video realistic dataset and the YCB-Video hybrid dataset. The AUC values of the five types of objects are plotted as a graph, as shown in Fig 4.

**Table 1:** Comparison of the experimental results of metrics testing between the real dataset and the hybrid dataset.

Categories	YCB-Video Reality Dataset		YCB-Video Hybrid Dataset	
	AUC	ADDpasstare	AUC	ADDpasstare
**003_cracker_box**	42.98%	86%	45.71%	93.3%
**005_tomato_soup_can**	18.75%	87.85%	28.06%	94.6%
**006_mustard_bottle**	28.46%	90.45%	35.58%	99.5%
**009_gelatin_box**	46.2%	100%	49.32%	100%
**010_potted_meat_can**	25.97%	89.9%	29.23%	90.2%
**mean**	32.47%	90.84%	37.58%	95.52%

As shown in Table 1, the hybrid dataset expanded using virtual reality is slightly better than the real dataset in terms of network prediction after 60 epochs of training. From Fig 4, when the threshold value is taken as 0.1 m, the pass rate of ADD improves by 4.68% to 95.52% from the average point of view, indicating the improved detection capability of the network. The AUC improved by 5.11%, indicating an increase in the pass rate at a fixed threshold, thus indicating a more refined detection of the network. The above comparison experiments demonstrate that the dataset built with virtual reality technology has the same validity as the known public dataset, eliminates the reprojection error caused by localization in the manual dataset and the pixel error in semantic segmentation, and its training effect is better.

### 2.3. The solution to the problem of occlusion of weakly textured parts

To improve the recognition rate of the occlusion problem, the dataset is processed to improve the resistance of the network to the occlusion situation. Compared to the LCHF algorithm [45], due to the small size of the artefacts, segmentation of the whole image may result in some images with complete artefact graphics and others without artefact graphics; this result is equivalent to only stitching the background without creating an occlusion situation. In this regard, this study proposes the following improvement by generating a local mask to mask only the position of the model. Specifically, read the part location information, generate a mask of random size, incomplete masking of the part, random masking of 0–80% of the surface area of the part, for the selection of the mask image should avoid the selection of pure colour block. This is because masking with a solid colour block may be treated as an artefact feature by the network and thus affect the subsequent processing. Therefore, the object is masked by randomly intercepting the background as a mask image, as shown in the following equation.

$$I_{h,w}=crop(Random(0,h-O_{h}),Random(0,w-O_{w}))$$ (3)

where *crop*() indicates image cropping, *h* indicates input image height, *w* indicates input image width, *O*_*h*_ indicates object height, *O*_*w*_ indicates object width, *Random*() generates random number, and *I*_*h*,*w*_ indicates the processed image, where the width and height of the processed image must satisfy the following equation:

$$\left\{ \begin{array}{l} 0\leq I_{h}\leq 0.8\times O_{h}\\ 0\leq I_{w}\leq 0.8\times O_{w} \end{array}\right.$$ (4)

**Fig 5:**
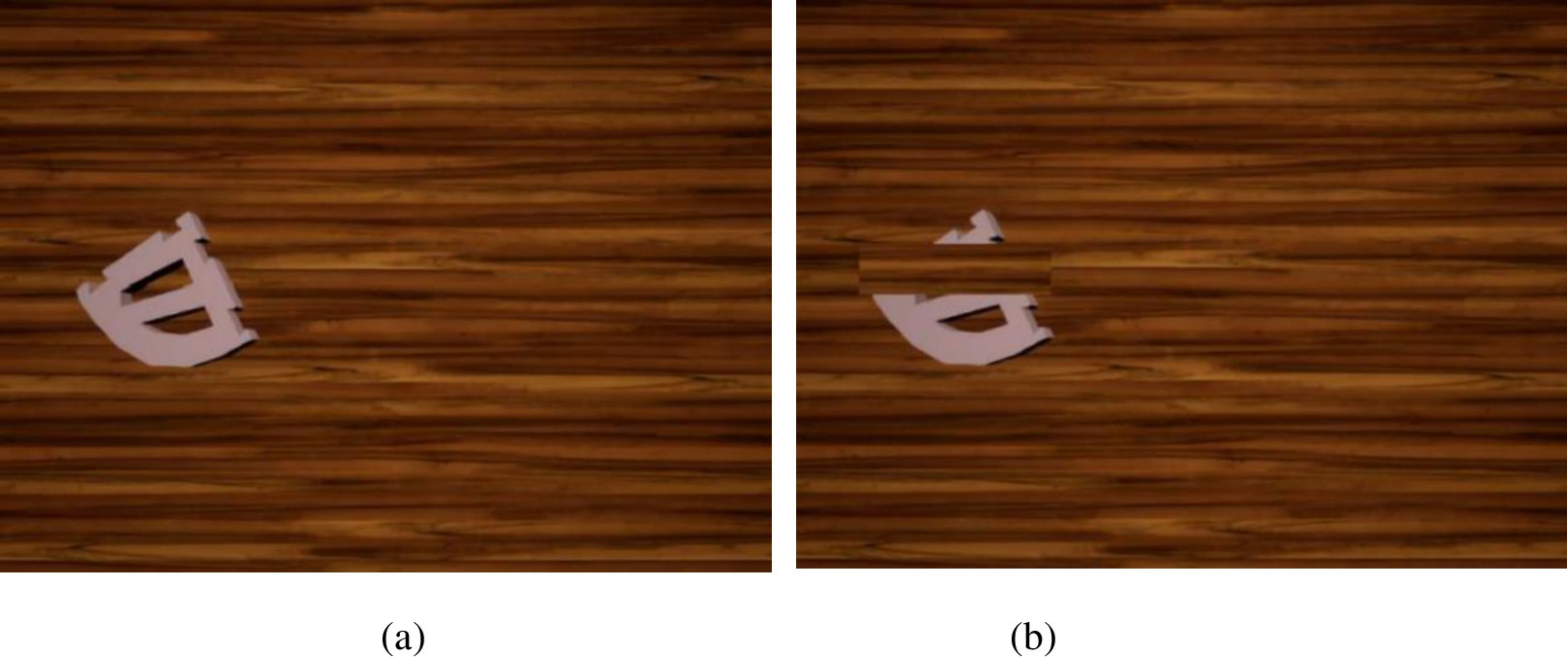
Local processing effect of random mask. (a) Before partial mask treatment. (b) After local mask treatment.

*I*_*h*_ denotes the height of the processed image and *I*_*w*_ denotes the width of the processed image. The results after random mask local processing are shown in Fig 5.

The DOPE++ network is trained on the weakly textured part dataset without local processing and the weakly textured part dataset with local processing and compared separately. The threshold value is taken as 0.05 m, the number of training iterations is 60, and the results of metrics testing on the validation set after training are shown in Table 2.

**Table 2:** Comparison of index test results with and without local treatment.

Categories	No treatment		Random Mask local processing	
	AUC	ADDpassrate	AUC	ADDpassrate
**Servoholder**	59.18%	84.05%	69.23%	92.35%
**PivConnector**	53.41%	86.35%	64.43%	91.75%
**Mean**	56.29%	85.2%	66.83%	92.05%

After 60 iterations of training the improved network on the random masked locally processed dataset, the ADD pass rate of the network improved, with a mean improvement of 6.85%. The mean improvement of the AUC metric was 10.54%, indicating an improvement in the accuracy of the locally processed network. When dealing with the actual occlusion situation, the above metrics only indicate that the network has no loss in accuracy and do not fully indicate whether the network has improved its resistance to the occlusion situation. In this paper, we designed a comparison experiment to test the ADD passing rate of the network without random mask local processing and the network with local processing by selecting 1000 images for each artefact and then applying 20%, 40%, 60% and 80% masking to each image. The experimental results are shown in Table 3.

**Table 3:** ADD pass rate at different shading rates.

Categories	Shading rate			
	20%	40%	60%	80%
**Unmasked trained Servoholder**	93.2	61.4	22.5	2.2
**Unmasked trained PivConnector**	92.2	60.8	24.3	0.3
**Masking trained Servoholder**	97.9	79.1	52.4	14.1
**Masking trained PivConnector**	94.7	76.6	50	10.5

As shown by the above experiments, the local masking of the dataset is beneficial to improve the resistance of the network to masking.

**Fig 6:**
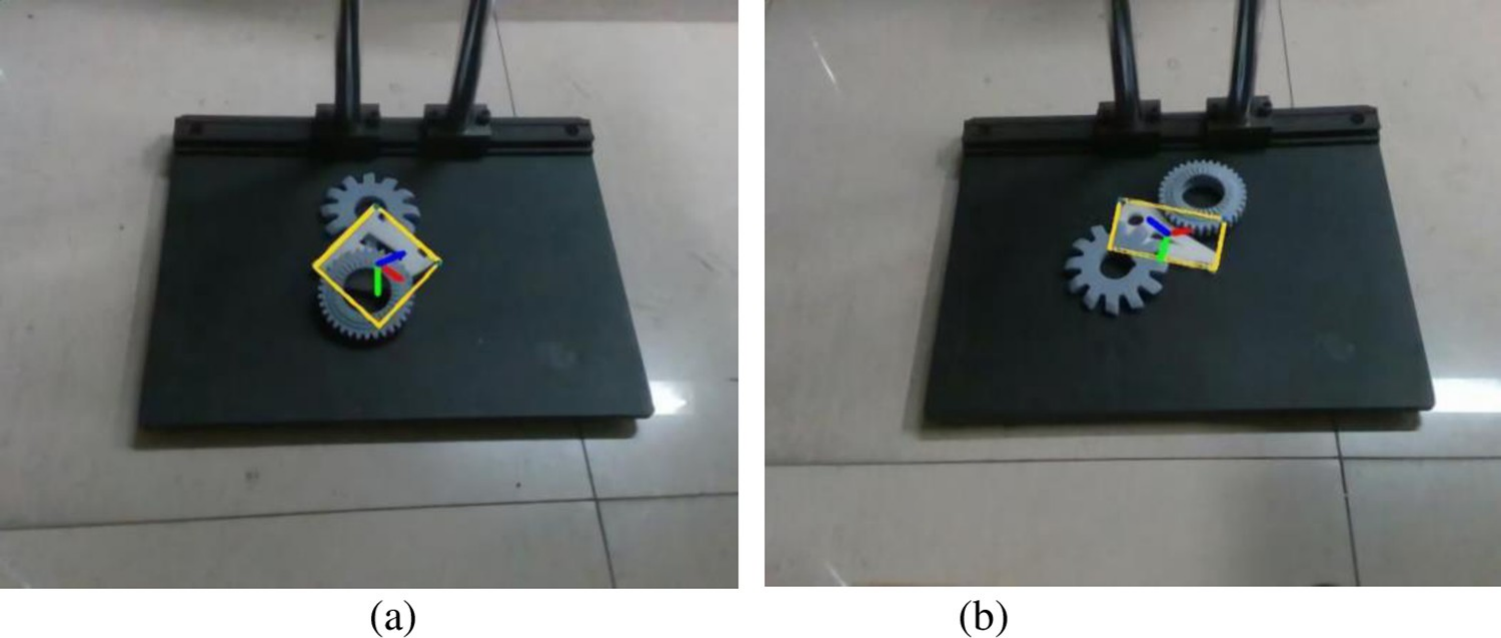
The actual detection effect in the case of occlusion. (a) PivConnector can be accurately detected even when obscured by interfering parts. (b) The Servoholder can be accurately detected even when obscured by interfering parts.

The above results are all verified in the hybrid dataset, and the detection effect for the masking situation during the actual detection is shown in Fig 6.

Fig 6 shows that both parts can still be accurately identified in the presence of interfering objects that obscure them. It can be concluded that our proposed random mask local processing method shows good resistance to the part occlusion problem.

## 3. Method

This section constructs the DOPE++ network by means of deep learning: a network for 6D pose estimation of weakly textured parts. The network proposes three improvements to the original DOPE network framework, including the incorporation of a depthwise separable convolution operation, an attention mechanism and a novel pose estimation module with multiscale feature fusion.

### 3.1. Lightweight feature extraction network based on depthwise separable convolution

**Fig 7:**
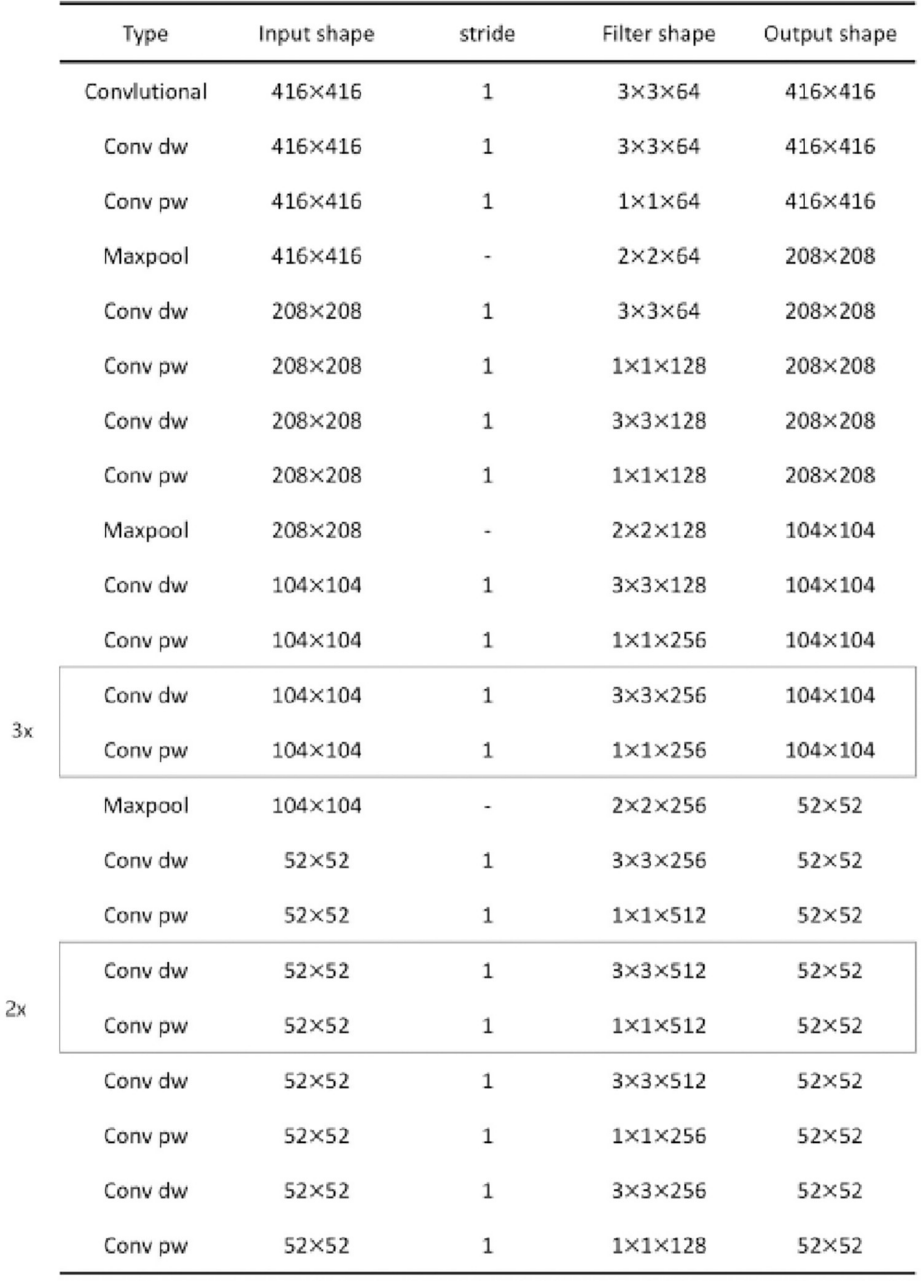
Backbone of the DOPE++ network. The backbone shows the structure of feature extraction of the DOPE++ network.

The original DOPE pose estimation network uses the first 24 layers of VGG19 [46] for feature extraction. In this paper, we introduce the depthwise separable convolution operation [47, 48] into the feature extraction part of the original DOPE network to improve the network operation speed. First, the depthwise conv operation is performed. When the input is a 3-channel RGB image, unlike the traditional convolution, which directly convolves three channels with three convolution kernels, only one convolution kernel is responsible for one channel, and the result is still a 3-channel feature map. Then, the pointwise conv operation is performed. The specific operation is to perform the convolution operation on all channels after the depthwise conv operation with the convolution kernel of the augmented dimension size number. The results of the operation are weighted and combined. The pointwise conv operation is similar to the traditional convolution operation, except that the size of the convolution kernel used in the pointwise conv operation is 1×1. The pointwise conv operation opens the connection between channels and completes the expansion of the feature dimension. The structure diagram of the improved feature extraction network is shown in Fig 7.

The results of the comparison between the improved feature extraction network parameters and the original feature extraction network parameters are shown in Table 4.

**Table 4:** Comparison of network parameters before and after improvement.

Network Name	Number of parameters
**Original feature extraction network**	9,696,958
**Improved feature extraction network**	1,106,696

As shown by the experimental results, the number of parameters of the improved network is approximately 1/9 of the original number of parameters, but the network structure remains unchanged.

### 3.2. Introduction of the attention mechanism

In this paper, we compensate for the loss of accuracy due to the reduction of network parameters and further improve the accuracy of the network by introducing an attention mechanism [49, 50]. First, the input features are filtered by average pooling and maximum pooling to produce two feature maps. Then, the two feature maps are imported into the two-layer MLP (multilayer perceptron) network, and the output features are subjected to an elemental intelligent summation operation, i.e., each given input vector is multiplied by the appropriate weight and then summed. The result is activated by the sigmoid activation function to obtain the channel attention feature map. The process is shown in the following equation [49].

$$M_{c}(F)=\sigma (MLP(AvgPool(F))+MLP(MaxPool(F)))$$ (5)

where *σ* denotes the activation function, *F* denotes the input features, and *M*_*c*_ denotes the channel attention feature map.

**Fig 8:**
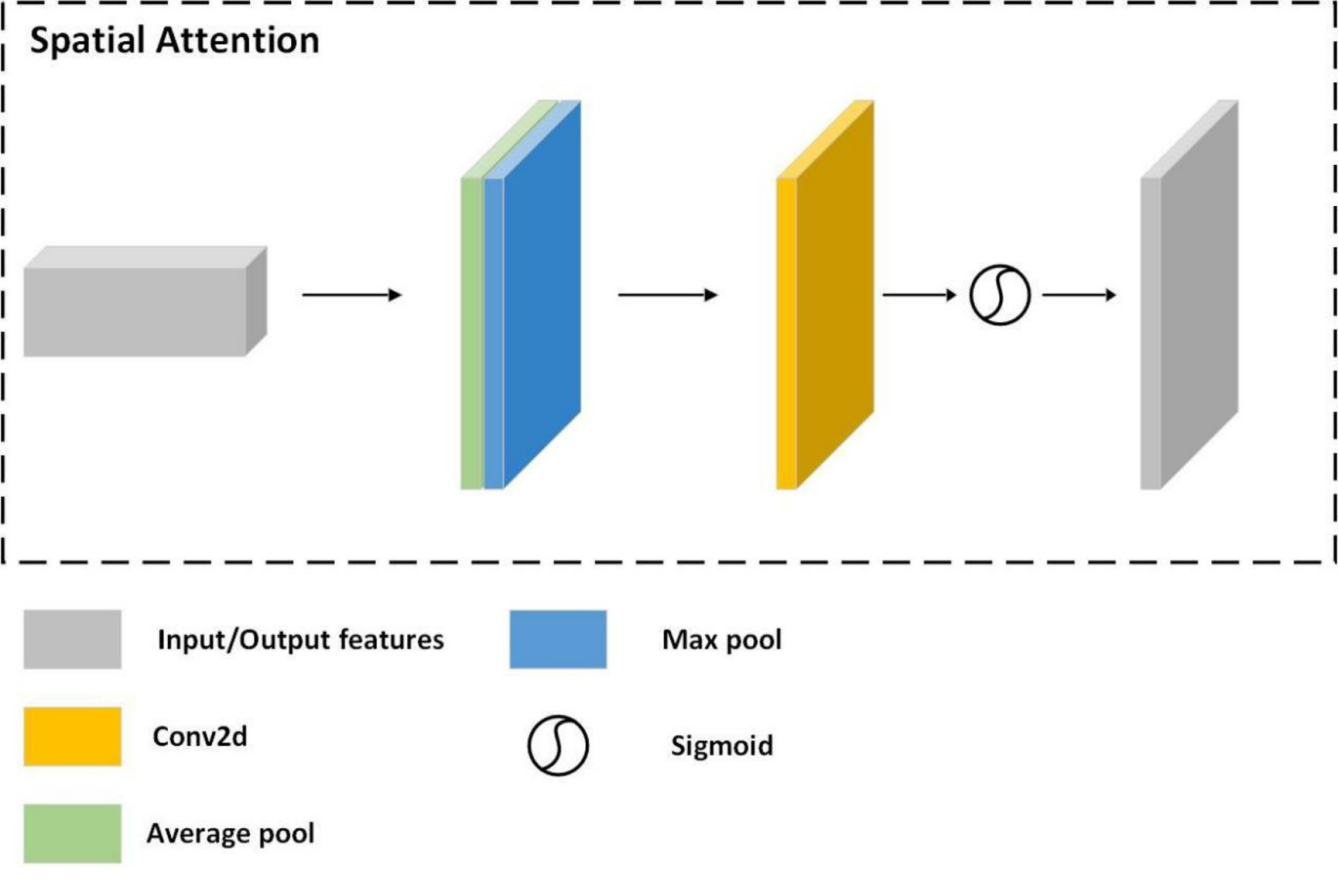
Spatial Attention structure diagram.

Next, the channel attention feature map is used as input to obtain the average pooling output and the maximum pooling output through the average pooling layer and maximum pooling layer, respectively. Then, the two outputs are stitched into a tensor and sent to a convolutional layer with a convolutional kernel size of 3x3. The result is activated with a sigmoid activation function to finally obtain the spatial attention feature map. The structure of the spatial attention module is shown in Fig 8.

The process is illustrated in the following equation [49].

$$M_{s}(F)=\sigma (f^{3\times 3}([AvgPool(F));MaxPool(F)])$$ (6)

where *σ* denotes the activation function, *F* denotes the input features, and *M*_*c*_ denotes the channel attention feature map.

**Fig 9:**
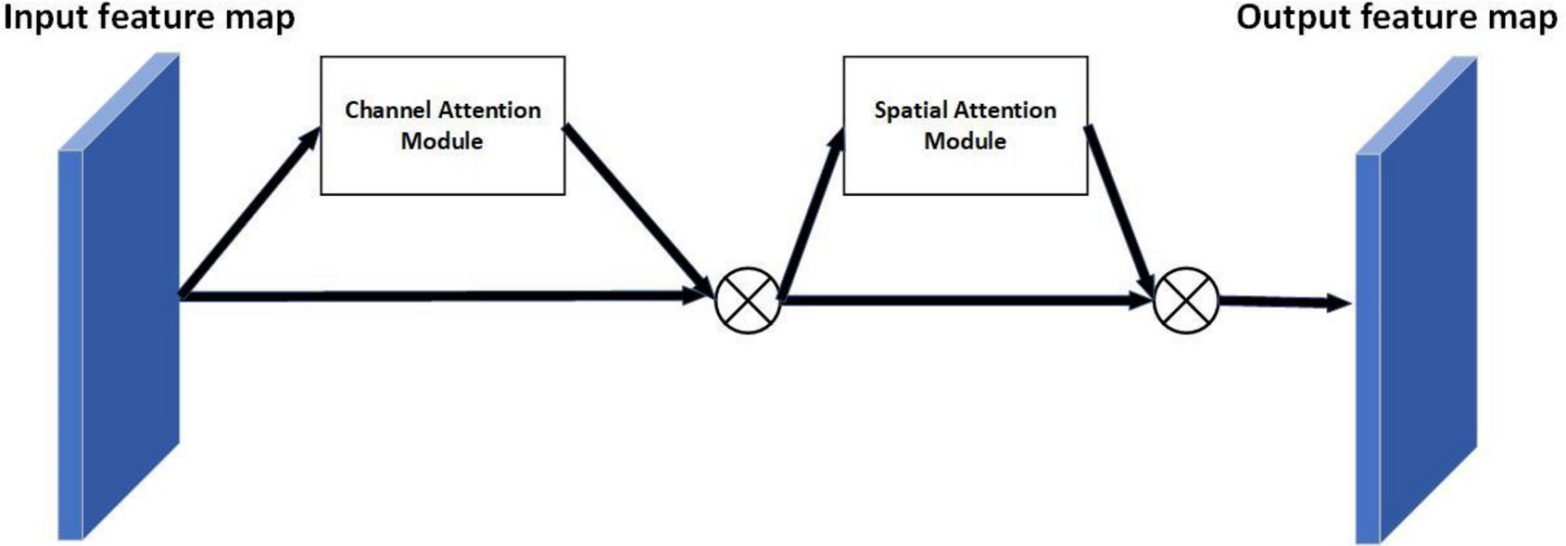
Overall structure of the attention module. We can substantially improve the accuracy of pose estimation with this attention module.

In this paper, the attention module is added to the layer after the feature extraction network; the overall module is shown in Fig 9.

### 3.3. Pose estimation network based on multiscale feature fusion

**Fig 10:**
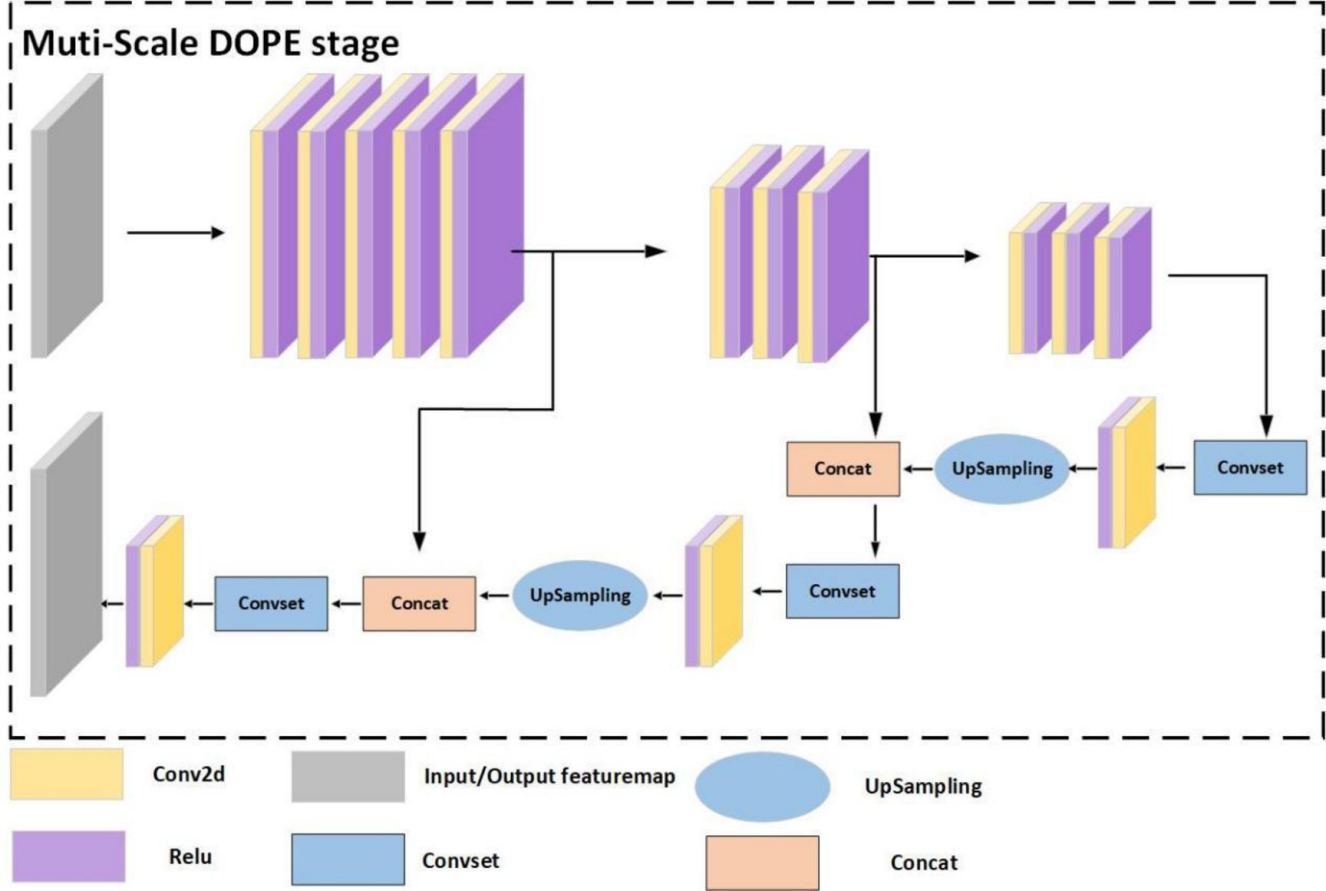
Structure diagram of the improved pose estimation module.

In this paper, the pose estimation module in the original network is improved by adding multiscale feature fusion [51, 52], which fuses feature maps of three different sizes. The improved pose estimation module is shown in Fig 10.

When the input feature dimension is 52×52×128, the feature map is first input into a five-layer convolution layer to obtain a feature map of size 52×52×9/16. The output result size is 52×52×9 when the prediction object is a confidence map; the output result size is 52×52×16 when the prediction object is a vector field. At this point, the output feature map size is 52, the input image size is 416, and the output is exactly 1/8 of the input. At this time, near the front end of the network, the perceptual field is relatively small, the feature map size is high, and the semantic representation of the feature map is weak, which is suitable for small target detection. The result is a feature map of size 26×26×9/16 by a three-layer convolutional layer; an output 1/16 of the input is suitable for the detection of medium-sized targets. Finally, the result is a three-layer convolutional layer to obtain a feature map of size 13×13×9/16, where the output is 1/32 of the input. This location is at the back end of the network, with a low feature map perceptual field relative to the larger feature map size, weak geometric representation and lack of spatial features, suitable for large target detection. The convolution set convset module consists of five layers of convolution and the ReLU activation function.

**Fig 11:**
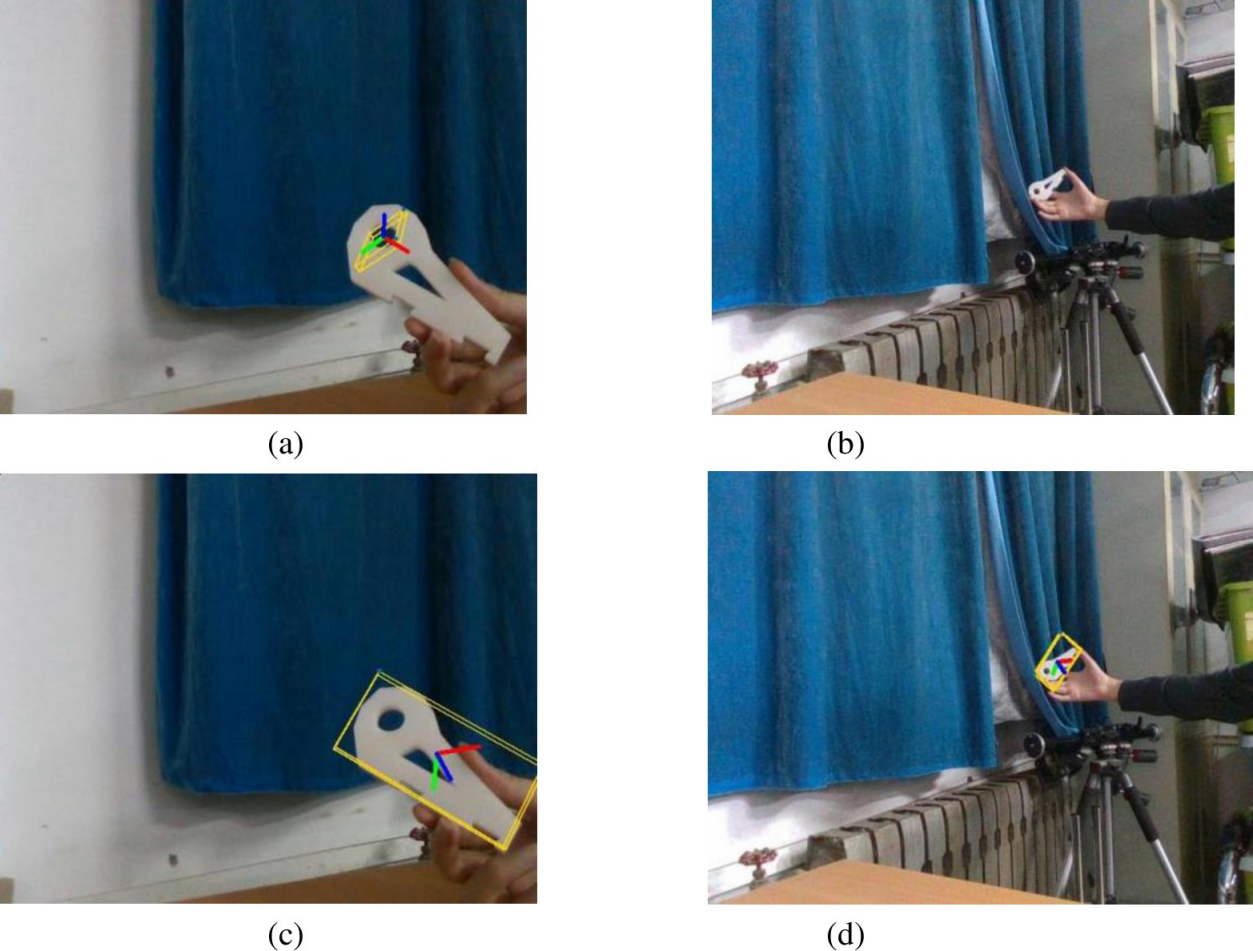
Comparison of the effect of recognizing parts of different scales before and after improvement. (a) DOPE network to identify large scale parts. (b) DOPE network to identify small-scale parts.(c) DOPE++ network to identify large scale parts.(d) DOPE++ network to identify small scale parts.

After obtaining feature maps at different scales at the front, middle and back of the improved pose estimation module, feature fusion is performed. The result of the feature map of size 13×13×9/16 is first imported into the convolutional set convset, which is 1×1,3×3,1×1,1×1,3×3 for each convolutional kernel layer. The result is then subjected to a convolution operation with a convolution kernel size of 1×1 and then 2-fold upsampling. The result is stitched with a feature map of size 26×26×9/16. Finally, through the same operation, the result is stitched with the feature map of size 52×52×9/16 the result is obtained by a convolution set and convolution operation to obtain the final prediction result. We call this improved module Multi-ScaleDOPEstage. The effect in the actual detection is shown in Fig 11.

As seen in Fig 11, the pose estimation network improved by multiscale feature fusion can detect objects of different sizes at different locations well, and the network’s ability to cope with detecting object scale changes has been greatly improved.

## 4. Experiments

In this section, we attempt to verify the effectiveness of our method. The DOPE algorithm has been shown to outperform networks such as PoseCNN, SSD-6D, and BB8. Therefore, the comparison of our approach with DOPE is sufficient to answer the question of whether our proposed network architecture is comparable to the latest technologies. In Section 4.1, we compare the proposed network with the original DOPE algorithm in three main aspects. Additionally, to further demonstrate the superiority of our algorithm, we compare it with state-of-the-art algorithms in recent years in Section 4.2. In Section 4.3, we build a vision-guided robot grasping platform to verify the feasibility of the algorithm for intelligent manufacturing.

### 4.1. Comparison with the DOPE algorithm

**Fig 12:**
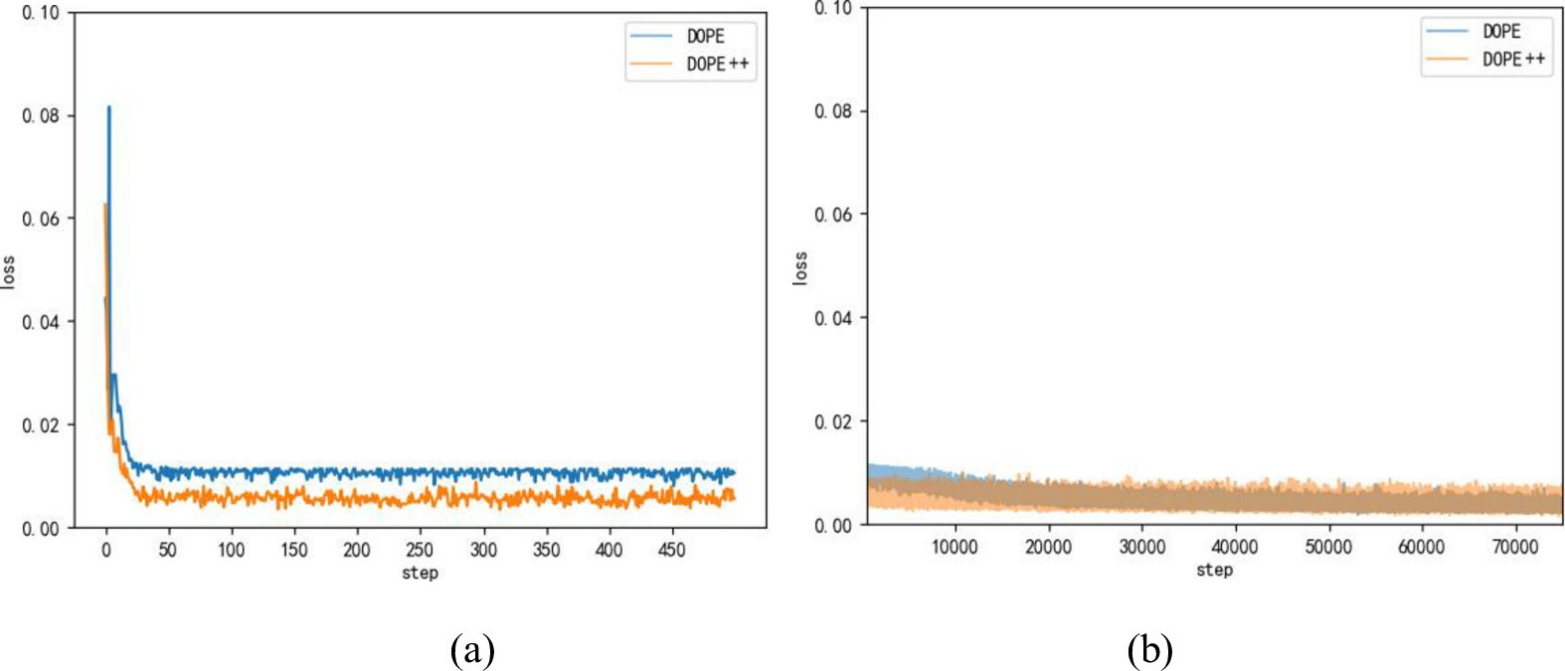
Comparison of loss curves before and after improvement. (a)The first 500 steps of the loss curve. (b) 500~75000 steps loss curve. The blue line represents the DOPE network and the orange line represents the DOPE++ network.

In this paper, three comparison experiments are designed. The network incorporating depthwise separable convolution, an attention mechanism, and multiscale feature fusion is referred to as the DOPE++ network (DOPE+DSC+attention+multiscale). The first experiment is a comparison experiment of the pose estimation accuracy of DOPE++ and DOPE. The loss curves of the DOPE network and the DOPE++ network are verified to determine the convergence ability of the DOPE++ network. A comparison of the training loss curves is shown in Fig 12.

From the comparison chart, we can see that the DOPE++ network has a loss value of approximately 0.06 at the beginning of training, while the DOPE network is approximately 0.08. Especially in the first 25 steps, the DOPE++ network converges faster and has a larger decreasing trend than the DOPE network. The loss value soon reaches approximately 0.007. The curvature change of the DOPE++ network levels off in 25–50 steps, while the DOPE network still has large fluctuations. In the subsequent training, the loss value of the DOPE network decreases slowly at 500~20,000 steps, and finally, the loss values of both networks are kept at approximately 0.003. By comparison, the DOPE++ network maintains a better level of convergence in the early stage of training, while the DOPE network converges more slowly at 30,000 steps before reaching convergence; the DOPE++ network converges more easily compared to the DOPE network.

**Fig 13:**
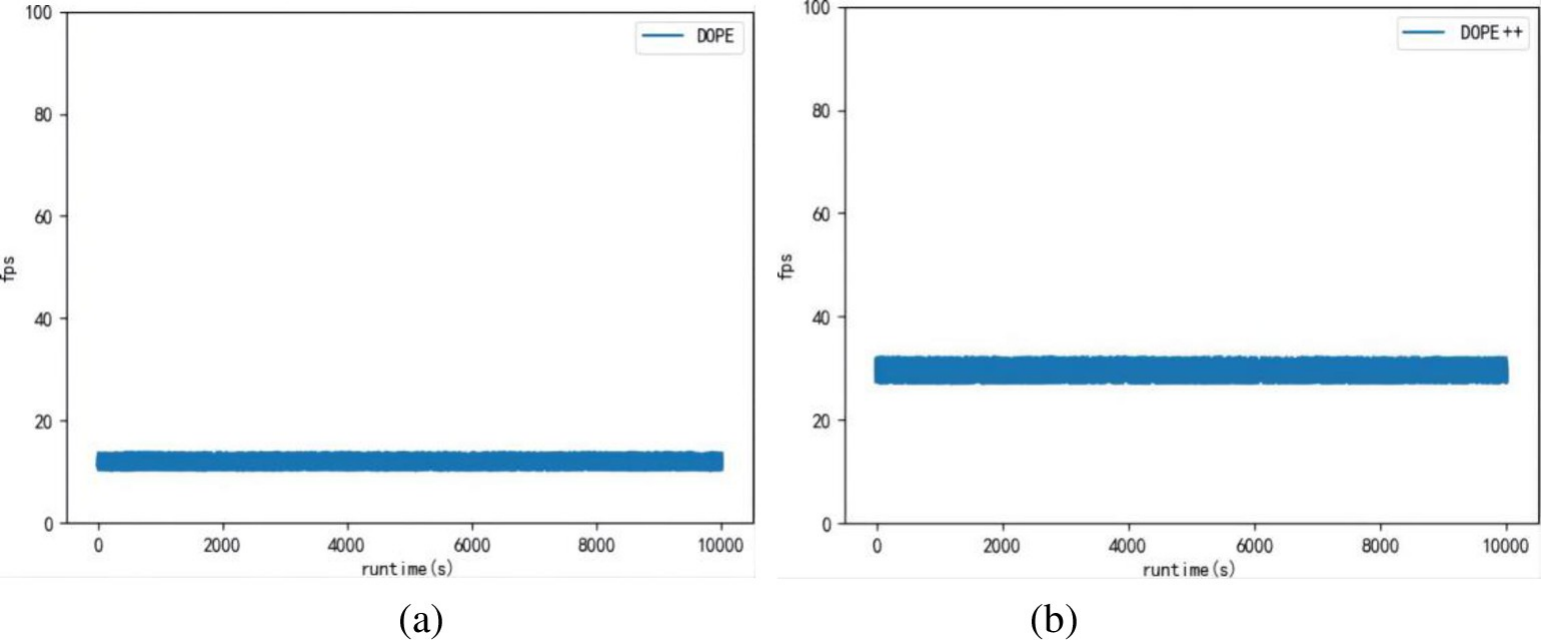
Frame rate graph of network operation before and after improvement. (a) Operating frame rate of the DOPE network.(b) Operating frame rate of the DOPE++ network.

The second experiment verifies that the DOPE++ network can significantly improve the detection frame rate compared to the DOPE network, thus meeting the real-time requirements. This experiment compares the frame rate perspective at runtime, reflecting the change in network detection efficiency after the light-weighting of the DOPE network. The comparison results are shown in Fig 13, where the x-axis is the runtime (in s) and the y-axis is the FPS.

The third experiment verifies that adding the attention mechanism can improve the accuracy of the network. The accuracy comparison between the DOPE+DSC *(DOPE network with depthwise separable convolution)* network and *DOPE+DSC+attention (not only the depthwise separable convolution operation but also the attention mechanism is introduced to the DOPE network)* network is performed to illustrate the compensation effect on accuracy loss after introducing the attention mechanism module. The evaluation metrics are ADD pass rate (threshold value of 0.05 m) and AUC area share. The experimental results are shown in Table 5.

**Table 5:** Comparison of the effect of pose estimation before and after the introduction of the attention mechanism.

Categories	DOPE+DSC		DOPE+DSC+Attention	
	AUC	ADDpassrate	AUC	ADDpassrate
**Servoholder**	42.28	79.1	48.82	81.15
**PivConnector**	45.14	82.2	52	87.55
**Mean**	43.71	80.65	50.41	84.35

From the experimental results, it can be concluded that the network accuracy of DOPE+DSC is effectively compensated for with the introduction of the attention mechanism.

Then, the comparison between the DOPE network and DOPE+DSC+attention+multiscale (Fusion of multiscale pose estimation modules on DOPE+DSC+attention network structure) network is performed, and the comparison results are shown in Table 6.

**Table 6:** Comparison of the effect of estimation before and after improvement.

Categories	DOPE		DOPE+DSC+Attention+Multi-Scale	
	AUC	ADDpassrate	AUC	ADDpassrate
**Servoholder**	46.51	83.45	51.71	87.65
**PivConnector**	48.25	88.10	59.56	94.40
**Mean**	47.38	85.77	55.63	91.02

From the experimental results, the improved network has an average improvement of 5.25% in ADD pass rate and 8.25% in AUC compared to the original network. It can be concluded that the improved scheme with the introduction of the attention mechanism and multiscale fusion has a significant improvement on the pose estimation accuracy of the network.

**Fig 14:**
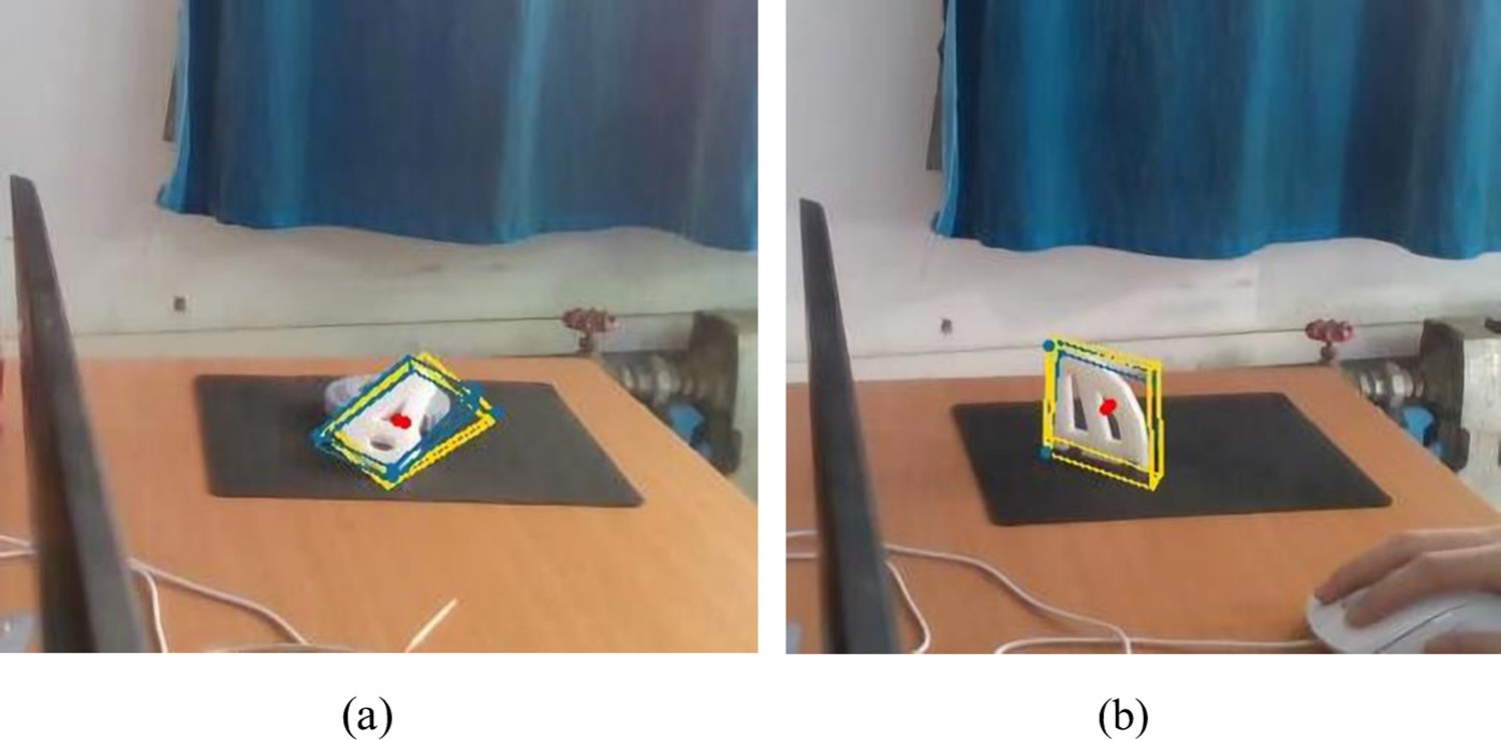
Comparison of pose estimation of DOPE network and pose estimation of DOPE++ network. (a) Servoholder. (b) PivConnector. Where the yellow wireframe is the minimum enclosing box of the DOPE++ network recognition result and the blue wireframe is the minimum enclosing box of the DOPE network recognition result.

Finally, the part pose recognition effect of the DOPE++ network and the DOPE network in actual operation is experimentally derived, and the comparison graph is shown in Fig 14.

As seen from the comparison results, the DOPE network pose estimation effect is relatively rough, and there are cases of pose estimation errors. The DOPE++ network pose estimation results are more refined, and the vertex prediction results are more accurate.

### 4.2. Comparison with state-of-the-art methods

Our approach is compared with state-of-the-art 6D pose estimation algorithms in recent years, including PoseCNN+ICP [20], DenseFusion [21], DeepIM [53], and DRNet [19].

Since most of the 6D pose estimation algorithms use the YCB-Video dataset, we use the objects in the YCB-Video dataset for 6D pose estimation validation. Table 7 shows the results of comparative experiments using our method and some state-of-the-art methods for 6D pose estimation for five objects in the YCB-Video dataset. Essentially, ADD is a better and more strict metric than ADD-S because it computes the distances between matched point pairs, which usually requires matches on both shape and texture. We therefore calculate the accuracy of ADD (<10%) for the current state-of-the-art method in Table 7. In Table 7, we find that our method is more accurate than the DenseFusion [21] algorithm that uses RGB-D images as input.

**Table 7:** Quantitative evaluation of pose estimation for the objects on the YCB-Video dataset.

Objects	PoseCNN+ICP	DenseFusion	DeepIM	DRNet	Our
**003_cracker_box**	73.3	98.2	83.6	74.28	93.3
**005_tomato_soup_can**	76.6	82.9	86.1	83.17	94.6
**006_mustard_bottle**	98.6	96.1	91.5	86.5	99.5
**009_gelatin_box**	100.0	100.0	71.9	89.36	100.0
**010_potted_meat_can**	77.9	79.8	76.2	79.16	90.2
**AVG**	85.28	91.4	81.86	82.494	95.52

Time efficiency is another important metric used to evaluate the usefulness of the algorithm. We tested the total pose estimation time of 0.033 s per frame on a GTX 1070 graphics card. See Table 8 for more time comparisons. Compared with DenseFusion [21] and DRNet [19], our method achieves the optimal time cost for 6D pose estimation to meet real-time requirements in manufacturing.

**Table 8:** Time efficiency evaluation for objects on the YCB-Video dataset.

	PoseCNN	DenseFusion	DRNet	Our
**Time (s)**	0.283	0.047	0.08	0.033

The above experimental results show that our method achieves the best current level in terms of accuracy and time efficiency.

### 4.3. Vision-guided robotic grasping system

**Fig 15:**
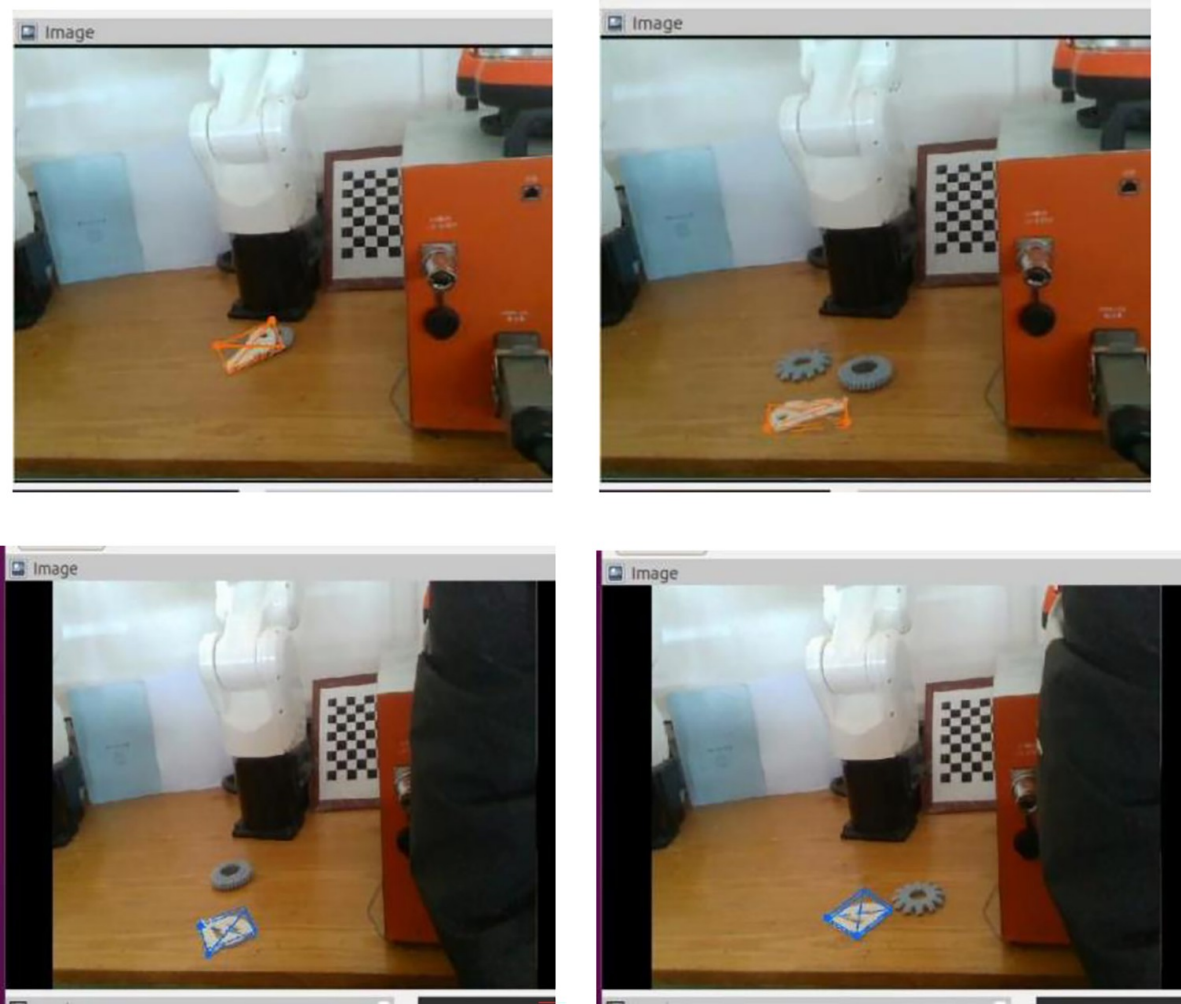
The effect of pose estimation when the robot gripping platform is actually operating.

In this paper, we build a weakly textured part pose estimation grasping platform in an experimental environment to simulate a real environment and verify the effectiveness of the proposed algorithm. The effectiveness of this algorithm is illustrated by actual repeated crawling experiments. In the vision part, a logitechC920e camera is used as the vision sensor, the robot is a Shenzhen Huacheng Industrial Control HC-S6 six-axis industrial robot, and the computer uses a GTX 1070 graphics card. This algorithm runs as a node on the ROS. Before repeating the gripping experiment, the bench calibration, tool calibration, hand-eye calibration and running the ROS system need to be performed sequentially. The experiment was performed in the laboratory with natural light without artificial light supplementation. In the experiments, first, the positional attitude of the part to be grasped is estimated through the network. Then, the pose in the camera coordinate system is converted to the pose in the robot arm coordinate system based on the conversion matrix calculation. Finally, the results are imported into the demonstrator for automatic path planning to capture weakly textured parts. If the capture is successful, it is called a pass. During the experiment, four groups of IMAGE display boxes were intercepted as recognition effect graphs; the network recognition effect is shown in Fig 15.

From the experimental results, it is seen that DOPE++ network recognition is more stable and can accurately detect two weakly textured parts (Servoholder and PivConnector). At the same time, there is no response to interference parts and good resistance to complex backgrounds. Then, 200 repetitive grasping experiments were conducted, and the image recognition success rate and robotic arm grasping success rate were counted separately every 50 steps. The test results are shown in Table 9.

**Table 9:** Statistical table of the results of 200 repeated crawls.

Number of catches	Number of successful recognition	Recognition success rate	Number of successful crawls	Crawl Success Rate
**1–50**	49	98%	47	94%
**51–100**	46	92%	46	92%
**101–150**	47	94%	43	86%
**151–200**	46	92%	45	90%
**Mean**	47	94%	45.25	90.50%

Data analysis was performed through comparative analysis methods. The data obtained from Table 7 show that the object recognition success rate is 92% in the interval of 51 to 100 catches. The object recognition success rate remains 92% in the interval of 151~200 catches. As the number of experiments increases, the success rate of object recognition does not fluctuate, which can indicate that the network is relatively stable.

## 5. Conclusions

We propose a 6D pose estimation method for weakly textured parts based on deep neural networks, which improves the accuracy and operation speed of 6D pose estimation. The experimental results show that our network runs at a frame rate better than 30 FPS and achieves an average accuracy of 91.02% when the threshold value is chosen as 0.05 m. At the same time, our proposed network has good resistance to the problems of occlusion and scale change that exist in practical engineering. In addition, we used virtual reality to build a simulation dataset of weakly textured parts. This method not only has a low production cost but also has a good training effect. In this paper, we contribute to the practical validation of 6D pose estimation of weakly textured parts by building a robot grasping simulation platform. The experimental results demonstrate the efficiency of our method, which is useful for real industrial scenarios. However, our method still has some limitations and performs poorly when dealing with pose estimation of symmetric objects, and the estimated object pose has a large error with the real position of the object.

In future work, we will continue to optimize the network to reduce the error between the pose estimation results and the true values and solve the 6D pose estimation problem for symmetric objects.
